# The Politics of Punishment: How Policy Shapes the Aging Experience in Prison and After Release

**DOI:** 10.1093/geroni/igaf122.197

**Published:** 2025-12-31

**Authors:** Kenzie Latham-Mintus, Raya Kheirbek, Elizabeth Vásquez

## Abstract

As the U.S. carceral system faces an unprecedented aging crisis, policies such as compassionate release, geriatric parole, and end-of-life care have emerged as critical yet underutilized mechanisms. This symposium explores how these policies profoundly influence the experiences of aging incarcerated individuals and those navigating reentry. Presentations will examine the systemic barriers to geriatric release and reentry, the ethical complexities of medical decision-making for incapacitated older adults in prison, and the privatization of prison hospice care. Additionally, this session will explore social vulnerability post-incarceration, analyzing how aging individuals experience social networks, social integration, and loneliness after release. By incorporating policy analysis, legal archival research, and a review of existing geriatric parole programs, this session highlights the urgent need for reform, advocacy, and interdisciplinary collaboration to address the health and social inequities faced by older adults in the carceral system.

